# Clinical and Microbiological Characteristics of Early *Candidozyma auris* Cases in a Tertiary Hospital in Istanbul, Türkiye: A Retrospective Case Series

**DOI:** 10.3390/antibiotics15090824

**Published:** 2026-08-25

**Authors:** Begüm Nalça Erdin, İrfan Aydın, Yüksel Akkaya, Meziyet Zeliha Gürlek Ustabaşı, Zeliha Alıcıkuş, Can Turan, İbrahim Halil Kılıç

**Affiliations:** 1 Department of Microbiology, Health Sciences University Darıca Farabi Training and Research Hospital, 41700 Kocaeli, Türkiye; begum.nalcaerdin@saglik.gov.tr; 2 Pharmacy Services Program, Pharmacy Services Department, Health Services Vocational School, Fenerbahce University, 34758 Istanbul, Türkiye; irfan.aydin@fbu.edu.tr; 3 Department of Microbiology, Health Sciences University Ümraniye Training and Research Hospital, 34764 Istanbul, Türkiye; yuksel.akkaya@sbu.edu.tr; 4 Department of Anesthesia and Resuscitation, Health Sciences University Ümraniye Training and Research Hospital, 34764 Istanbul, Türkiye; m.gurlekustabasi@saglik.gov.tr (M.Z.G.U.); zeliha.alicikus@sbu.edu.tr (Z.A.); 5 General Intensive Care Unit, Acıbadem Ataşehir Hospital, 34642 Istanbul, Türkiye; can.turan@acibadem.com; 6 Department of Biology, Faculty of Arts and Sciences, Gaziantep University, 27310 Gaziantep, Türkiye

**Keywords:** *Candidozyma auris*, *Candida auris*, antifungal resistance, candidemia, intensive care unit, Türkiye, retrospective case series

## Abstract

Background: *Candidozyma auris* (*C. auris*) is an emerging multidrug-resistant yeast associated with healthcare-related transmission, difficult laboratory identification, antifungal resistance, and high mortality in critically ill patients. This study aimed to describe the clinical and microbiological characteristics of early institutional *C. auris* cases detected in a tertiary hospital in Istanbul, Türkiye. Methods: Adult patients hospitalized between January 2021 and December 2022 with *C. auris* isolation were retrospectively reviewed. Demographic data, underlying conditions, intensive care unit (ICU) exposure, invasive procedures, antimicrobial and antifungal use, culture sources, antifungal susceptibility testing (AFST) results, and clinical outcomes were evaluated. *C. auris* isolates were identified using VITEK MS v.3.2, and AFST was performed using *C. auris* isolates were identified using VITEK MS v.3.2, and AFST was performed using Sensititre YeastOne YO10 (Thermo Fisher Scientific, Waltham, MA, USA) panels. Results were interpreted according to CDC interim breakpoints. Multiple isolates from an individual patient were retained when they represented different specimen types or sampling events. Results: Thirty-three adult patients and 62 clinical isolates were included. The mean age was 67.8 years, and 20 patients (60.6%) were aged 65 years or older. Most patients had prior ICU admission (93.9%), urinary catheterization (97.0%), central venous catheterization (90.9%), mechanical ventilation (81.8%), and broad-spectrum antimicrobial exposure. Blood cultures represented the most frequent source of isolation (56.5%), followed by urine cultures (27.4%). Resistance rates were 98.4% for amphotericin B, 88.7% for fluconazole, 6.6% for micafungin, and 6.5% for caspofungin. Overall mortality was 72.7%. While mortality was numerically higher among patients aged 65 years or older and those requiring inotropic support, only the need for mechanical ventilation was statistically significantly associated with mortality. The cohort-level APACHE II-based expected mortality was 48.0%, whereas observed mortality was 72.7%; this comparison was descriptive and no formal calibration analysis was performed. Conclusions: Early institutional *C. auris* cases in this tertiary hospital occurred predominantly among elderly, critically ill patients with prolonged healthcare exposure, invasive devices, and extensive antimicrobial use. The findings support the need for early laboratory recognition, cautious interpretation of commercial AFST results, particularly given the propensity of Sensititre YeastOne to overestimate amphotericin B resistance, strengthened infection control practices, and multicenter studies incorporating molecular typing to clarify transmission dynamics and antifungal resistance patterns.

## 1. Introduction

*Candidozyma auris* (*C. auris*), formerly referred to as *Candida auris*, is a multidrug-resistant (MDR) yeast that has rapidly become a serious global public health threat. *C. auris* can cause life-threatening invasive infections, particularly in hospitalized and critically ill patients, and reported mortality rates generally range between 29% and 53%, reaching up to 70% in some settings [1,2,3]. Although several aspects of *C. auris* epidemiology remain incompletely understood, advanced age, prolonged intensive care unit (ICU) stay, exposure to broad-spectrum antibacterial and antifungal agents, invasive medical devices, and underlying comorbid diseases are recognized risk factors for colonization and infection [3,4].

In Türkiye, the first reported *C. auris* case was described in Istanbul in 2020 [5]. The Ministry of Health published a national document in 2025 stating that suspected *C. auris* isolates began to be submitted from hospitals in June 2020; however, the report provides data only for a three-month period in 2024 [6]. Published studies from Türkiye covering 2020 and 2021 generally included small patient numbers or single-center experiences; except for one case reported from İzmir, all early cases were reported from Istanbul [7,8,9,10]. In our hospital, which serves approximately 3 million patients annually in Istanbul, the first *C. auris* isolate was detected in April 2021, followed by an increase in cases during 2022. Early institutional data are important because they document the clinical setting in which this pathogen emerged, identify high-risk patient groups, and help guide local infection control and antifungal stewardship strategies.

This retrospective study aimed to describe the demographic, clinical, epidemiological, and microbiological characteristics of adult patients with early *C. auris* isolation in our hospital between 2021 and 2022. In the absence of molecular typing, this study cannot definitively confirm clonal transmission. Instead, it characterizes the spatiotemporal clustering of early institutional cases to provide a clinical and microbiological foundation, highlighting areas that necessitate strengthened surveillance and future molecular epidemiological investigations.

## 2. Results

### 2.1. Demographic and Clinical Characteristics of the Patients

Of the 33 adult patients, 14 (42.4%) were male and 19 (57.6%) were female. Ages ranged from 29 to 95 years, with a mean age of 67.8 years; 20 patients (60.6%) were aged 65 years or older. The most common reasons for admission were sepsis (n = 13, 39.4%), respiratory system diseases (n = 11, 33.3%), and postoperative complications (n = 7, 21.2%). Approximately 70% of the patients had two or more comorbid diseases. Hypertension (n = 20, 60.6%) and diabetes mellitus (n = 13, 39.4%) were the most common comorbidities, followed by acute or chronic renal failure, cerebrovascular events, and malignancies. At the time of first *C. auris* isolation, 13 patients (39.4%) were in the anesthesia ICU (AICU), 16 (48.5%) were in the internal medicine ICU (IMICU), and 4 (12.1%) were in the palliative care unit (PCU) (Table 1).

**Figure 1:**
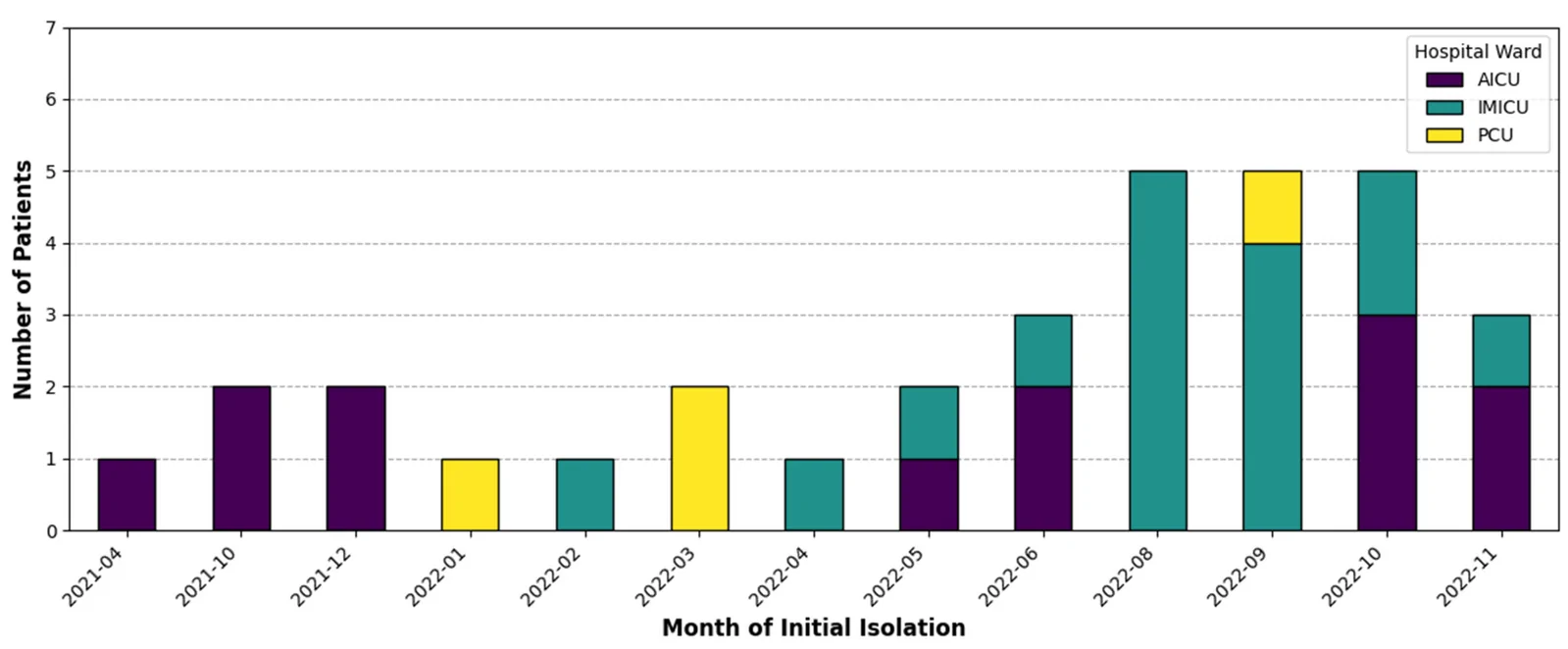
Epidemic curve of early *Candidozyma auris* cases based on initial isolation dates (n = 33). To avoid analytical unit duplication, only the first positive culture for each patient is included. The graph highlights the clustering of cases, predominantly within the intensive care units during the autumn months of 2022. In the absence of molecular typing, this pattern illustrates spatiotemporal clustering rather than definitive healthcare-associated transmission. AICU: Anesthesia Intensive Care Unit; IMICU: Internal Medicine Intensive Care Unit; PCU: Palliative Care Unit.

Five patients had their first *C. auris* isolation in 2021 and 28 in 2022. The first institutional case was detected in April 2021, followed by a marked increase in 2022. At the time of first isolation, 16 patients were in the IMICU, 13 in the AICU, and 4 in the PCU. Fifteen first isolations (45.4%) occurred during autumn (*p* = 0.039). Figure 1 presents the patient-level epidemic curve by initial isolation date and hospital unit. In the absence of molecular typing, these observations represent spatiotemporal clustering rather than proof of healthcare-associated or clonal transmission.

### 2.2. Invasive Procedures, Therapeutic Interventions, and Other Risk Factors

Before the first *C. auris* isolation, 31 patients (93.9%) had at least one ICU admission, including 22 patients with one ICU admission and 9 patients with two or more ICU admissions. Only 2 patients (6.1%) had no prior ICU admission.

The total LOS ranged from 7 to 205 days, with a mean of 72.73 ± 54.70 days. Urinary catheters were present in 97.0% of patients, central venous catheters in 90.9%, mechanical ventilation in 79.0%, and at least one inotropic agent was required in 72.7% of patients.

All patients received broad-spectrum antibacterial treatment before *C. auris* isolation. Five patients (15.2%) received one antimicrobial class, 9 patients (27.3%) received two classes, and 19 patients (57.6%) received three or more classes. Meropenem was the most frequently used antibacterial agent (29/33, 87.9%), followed by teicoplanin (16/33, 48.5%) and polymyxin-group agents (15/33, 45.5%) (Table 2).

Regarding antifungal treatment, 12 patients received a single antifungal agent, 11 received two different antifungal agents, and 3 received three different antifungal agents. Seven patients did not receive antifungal therapy; four of these patients died before treatment could be initiated. Fluconazole (42.4%) and micafungin (33.3%) were the most commonly used antifungal agents. For treated patients, the exact time from culture positivity to antifungal initiation and the classification of therapy as empirical or targeted were not consistently available in the retrospective records; therefore, treatment timeliness and appropriateness could not be evaluated reliably. The complete patient-level demographic, clinical, intervention, and outcome dataset is provided in Table S1.

### 2.3. Microbiological Characteristics of the Isolates

**Figure 2:**
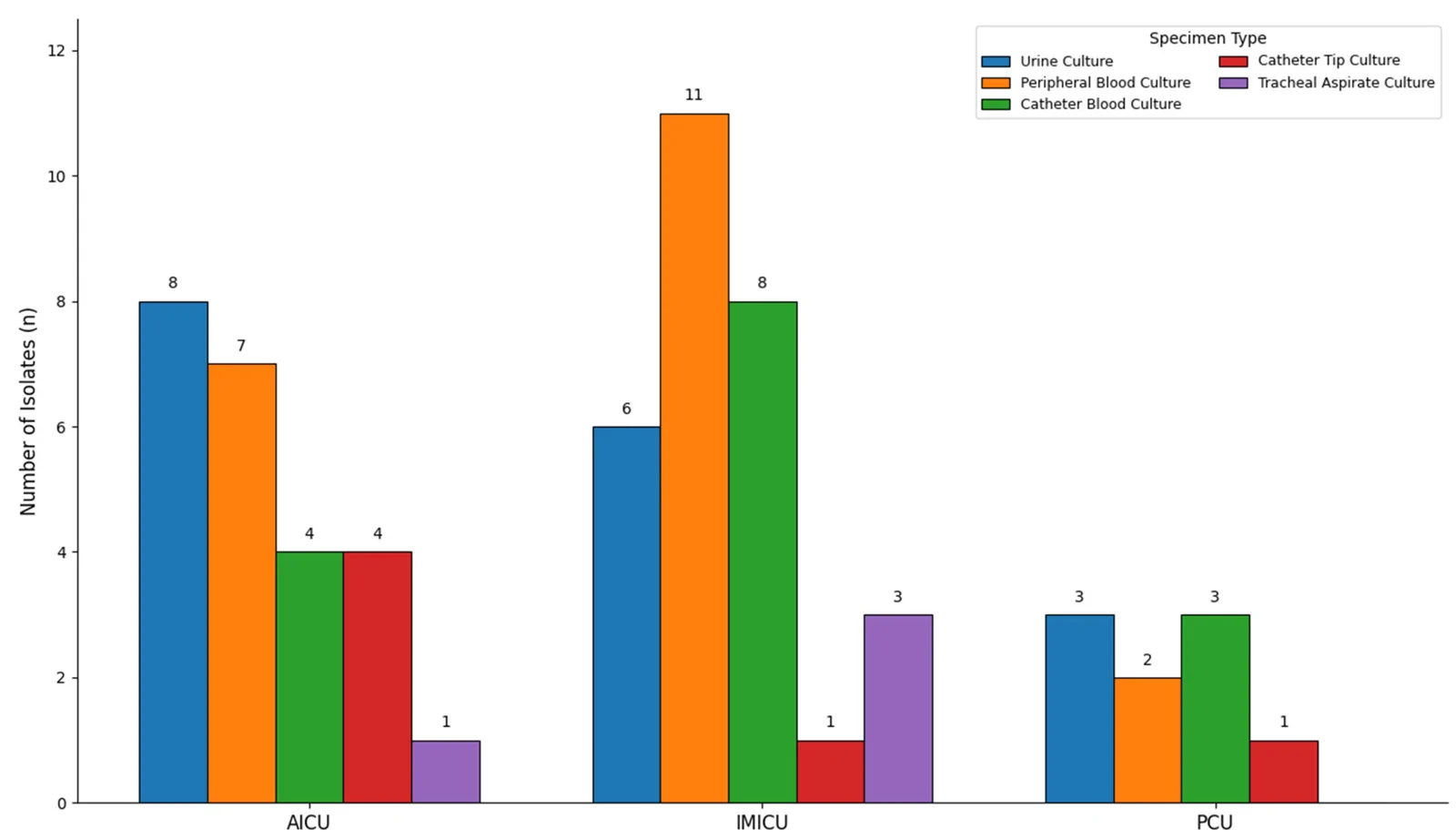
Distribution of *C. auris* isolates by specimen type and hospital ward (isolate-level analysis, n = 62). Blood cultures accounted for 35/62 isolates (56.5%), followed by urine cultures (17/62, 27.4%), catheter-tip cultures (6/62, 9.7%), and tracheal aspirate cultures (4/62, 6.5%). AICU: Anesthesia Intensive Care Unit; IMICU: Internal Medicine Intensive Care Unit; PCU: Palliative Care Unit.

A total of 62 clinical *C. auris* isolates obtained from 33 patients were included in the microbiological analysis. According to hospital ward, 24 isolates (38.7%) were recovered in the AICU, 29 (46.8%) in the IMICU, and 9 (14.5%) in the PCU. According to specimen type, 35 isolates (56.5%) were recovered from blood cultures, including 20 peripheral blood cultures (32.3%) and 15 catheter blood cultures (24.2%). Urine cultures accounted for 17 isolates (27.4%), catheter-tip cultures for 6 (9.7%), and tracheal aspirate cultures for 4 (6.5%) (Figure 2).

The observed antifungal resistance rates were 98.4% for amphotericin B, 88.7% for fluconazole, 6.6% for micafungin, and 6.5% for caspofungin (Table 3). The amphotericin B estimate should be interpreted cautiously because Sensititre YeastOne may overestimate amphotericin B MICs and resistance in *C. auris*. The complete isolate-level antifungal susceptibility profiles and MIC data are provided in Table S2.

### 2.4. Outcome Analysis

The overall mortality rate was 72.7% (n = 24). Due to the limited sample size and the retrospective nature of the study, mortality-related findings were considered descriptive and exploratory. The mean age of non-survivors (69.0 ± 19.9 years) was higher than that of survivors (64.8 ± 12.7 years), but the difference was not statistically significant (*p* > 0.05). Mortality was numerically higher among patients aged 65 years or older (80.0%, 16/20) than among those younger than 65 years (61.5%, 8/13), but the difference was not statistically significant (*p* = 0.425). Mortality was 64.3% among males and 78.9% among females, with no statistically significant difference between sexes (*p* = 0.442).

Mortality was higher among patients with three or more comorbid diseases (80.0%, 12/15) than among those with fewer comorbidities (66.7%, 12/18), but the difference was not statistically significant (*p* = 0.458). The presence of multiple comorbidities was not significantly associated with LOS (*p* = 0.704).

In the exploratory univariable analysis, mechanical ventilation was associated with mortality (*p* = 0.034). Inotropic support was required in 83.3% of non-survivors (20/24) and 44.4% of survivors (4/9), but this difference was not statistically significant (*p* = 0.073). LOS was longer among survivors (*p* = 0.041), a finding that should be interpreted cautiously because early death may shorten total hospitalization time. These associations likely reflect baseline illness severity and multiorgan dysfunction in a critically ill ICU population rather than an independent prognostic effect of *C. auris*.

Mortality did not differ significantly between patients with candidemia (72.0%, 18/25) and those without candidemia (75.0%, 6/8; *p* = 1.000). Mortality was 61.9% (13/21) among patients treated with echinocandins, 100% (5/5) among those treated with fluconazole and/or amphotericin B, and 85.7% (6/7) among patients who received no antifungal therapy; the overall difference was not statistically significant (*p* = 0.280) (Table 4).

Baseline APACHE II scores did not differ significantly between operative and non-operative patients (25.3 ± 5.9 vs. 25.1 ± 4.3, respectively; *p* = 0.815). APACHE II-based expected mortality was also not significantly different between the groups (42.2 ± 25.1% vs. 49.3 ± 14.1%; *p* = 0.300). Observed mortality was 50.0% (3/6) among operative patients and 77.8% (21/27) among non-operative patients (*p* = 0.309). In the overall cohort, mean APACHE II-based expected mortality was 48.0 ± 16.4%, whereas observed mortality was 72.7% (24/33). Given the limited sample size and the absence of formal calibration analysis, these findings were considered descriptive and exploratory (Table 5).

## 3. Discussion

This retrospective institutional case series describes the clinical and microbiological characteristics of early *C. auris* cases detected in a tertiary hospital in Istanbul during 2021–2022. The main findings were that *C. auris* occurred predominantly in elderly, critically ill patients with prolonged healthcare exposure, frequent ICU admission, invasive devices, and extensive broad-spectrum antimicrobial use. Bloodstream isolation was the most common microbiological presentation, antifungal resistance to fluconazole was frequent, amphotericin B resistance appeared very high by the commercial method used, and overall mortality was high. However, mortality-related findings should be interpreted in the context of severe underlying illness and the small sample size.

Monitoring and reporting early institutional *C. auris* cases are important for strengthening diagnostic microbiology capacity, infection control measures, and antifungal stewardship. Although reports from Türkiye have increased, early publications from 2020 and 2021 generally included small numbers of cases [5,7,8,9,10]. In our hospital, the first *C. auris* case was detected on 29 April 2021 in the AICU from the blood culture of a 91-year-old female patient on the 51st day of hospitalization. After five cases in 2021, an increase was observed in 2022, especially in ICU-related settings. These data suggest institutional clustering in time and place; however, because molecular typing was not performed, the findings cannot confirm clonal transmission, a single source, or a specific transmission chain. This distinction is important because time-unit clustering alone is insufficient to establish molecular epidemiological relatedness.

Molecular studies reported from Türkiye have so far indicated a predominance of the South Asian lineage (Clade I) among characterized isolates. Whole-genome sequencing of 10 isolates from a COVID-19 ICU cluster in Istanbul showed the closest similarity to Clade I [8]. In a later study from our institution, 57 isolates were classified as Clade I using a commercial clade-detection real-time PCR assay according to the manufacturer’s interpretation [11]. More recently, a multicenter study from Türkiye characterized 47 clinical isolates as Clade I using sequencing-based methods [12]. These findings suggest that Clade I is the predominant lineage among Turkish isolates investigated molecularly to date. However, the isolates in the present 2021–2022 cohort were not typed; therefore, their clade assignment and genetic relatedness cannot be inferred from the temporal or ward distribution.

The mean age of the patients was 67.8 years, and 60.6% were aged 65 years or older. Previous studies from our hospital reported that 65.9% of 31 patients in 2022–2023 were older than 65 years and that most patients in 2023–2024 were older adults, supporting the continued predominance of *C. auris* among older hospitalized patients [11,13]. Advanced age is a well-established risk factor for *C. auris* infection, and the early and later institutional data from our hospital are consistent with this pattern [14,15]. Although male sex is often reported as a risk factor in the literature, 57.6% of patients in our cohort were female. Similar observations have been reported in some studies, but this finding requires further evaluation in larger datasets [15].

The most common admission diagnoses were sepsis, respiratory system diseases, and postoperative complications. Most patients had multiple comorbidities, particularly hypertension and diabetes mellitus. These findings are consistent with national and international reports showing that *C. auris* primarily affects patients with severe underlying diseases and prolonged healthcare exposure [6,16]. At the time of first isolation, most patients were in ICUs, and nearly all had prior ICU exposure. This supports the role of ICU environments as important settings for *C. auris* detection and possible persistence, although environmental sampling and molecular comparison would be required to demonstrate environmental reservoirs or transmission pathways.

Invasive procedures and antimicrobial exposure were highly prevalent in this cohort. Urinary catheters, central venous catheters, mechanical ventilation, and inotropic support were common, and all patients had received broad-spectrum antibacterial therapy. Meropenem was the most frequently used antibacterial agent. These findings overlap with CDC guidance and international reports identifying healthcare exposure, invasive devices, and broad-spectrum antibiotic use as major risk factors for *C. auris* colonization and infection [16,17]. They also highlight the importance of device management, antimicrobial stewardship, and strict infection prevention measures in high-risk units such as ICUs.

The mean LOS was 72.73 days, which appears longer than values reported in some international cohorts [18]. Prolonged hospitalization is a recognized risk factor for *C. auris* acquisition, but in our study LOS was significantly longer among survivors than non-survivors. This apparently contradictory finding should not be interpreted as a protective effect of prolonged hospitalization; rather, early death may have shortened LOS among non-survivors. Additionally, ICU bed utilization dynamics and delayed discharge for non-infectious reasons may influence LOS in Türkiye [19]. Therefore, LOS-related findings should be considered descriptive and context-dependent.

An increase in case detection was observed during autumn. A later study from our hospital also reported that approximately 53% of cases in 2022–2023 occurred in autumn [13]. However, the Ministry of Health report described a fluctuating pattern rather than a consistent seasonal trend [6]. Although seasonal variation has been described for some Candida infections, definitive evidence for seasonality in *C. auris* infections remains limited [20,21,22]. Larger multicenter studies are required to clarify whether seasonal variation reflects true biological patterns, healthcare system factors, or local outbreak dynamics.

Blood cultures were the most common source of *C. auris* isolation in our cohort, followed by urine cultures. Similar patterns were reported in later studies from our hospital and in national surveillance data, where bloodstream infections constituted the major clinical presentation [6,11,13]. This finding underscores the clinical importance of *C. auris* in invasive disease and the need for rapid identification of Candida isolates recovered from blood cultures, particularly in critically ill patients.

AFST interpretation deserves particular caution. During the study period, definitive EUCAST breakpoints for *C. auris* were not yet available, and the results were interpreted using CDC interim breakpoints and the commercial SYO method [17]. EUCAST breakpoints were later established in 2025, with recommendations emphasizing standardized broth microdilution testing. In this cohort, resistance rates were high for amphotericin B and fluconazole, whereas echinocandin resistance was uncommon. The very high amphotericin B resistance rate should be interpreted carefully because commercial methods, including Sensititre YeastOne, may overestimate amphotericin B resistance in *C. auris* [23,24]. Similar variability in amphotericin B susceptibility has been reported in studies from Türkiye and elsewhere, emphasizing the need for standardized and comparable AFST approaches [11,12,13,25,26,27].

Although fluconazole MIC values were high, voriconazole and posaconazole MIC values were relatively low in our dataset. This observation is consistent with the CDC guidance that fluconazole-resistant isolates may sometimes show lower MICs to other azoles and that treatment decisions should be individualized based on the full susceptibility profile and clinical context. Nevertheless, echinocandins remain important therapeutic options, and the detection of micafungin and caspofungin resistance in urine isolates is noteworthy. The clinical significance of these findings should be interpreted cautiously because urine isolates may represent colonization rather than invasive infection.

Seven patients did not receive antifungal treatment, and four of these patients died before treatment could be initiated. This finding emphasizes the clinical importance of timely diagnosis and early communication between the microbiology laboratory and treating clinicians. Fluconazole and micafungin were the most commonly used antifungals. Mortality appeared lower among patients treated with echinocandins than among those who received fluconazole/amphotericin B or no antifungal therapy, but this difference was not statistically significant. Given the high fluconazole resistance rate, the selection of antifungal treatment in ICU patients should be guided by rapid identification, AFST results, local resistance patterns, and current treatment recommendations.

Overall mortality was 72.7%, which is high compared with many reported *C. auris* cohorts but within the upper range described in critically ill populations [3,28]. While mechanical ventilation was statistically associated with mortality, the numerical trends observed for advanced age and inotropic support did not reach statistical significance. These findings likely reflect the profound severity of underlying illness and multiorgan dysfunction in a critically ill population rather than an independent prognostic effect of *C. auris*. The mean APACHE II-based expected mortality for the cohort was 48.0%, whereas observed mortality was 72.7%; the descriptive difference was most pronounced in non-operative patients (49.3% expected vs. 77.8% observed). This observation may indicate that a baseline severity score alone does not fully capture dynamic clinical and infection-specific factors. However, because of the small sample size and the absence of formal calibration or discrimination analyses, the present study does not establish the prognostic performance of APACHE II in *C. auris* infection [29].

Because this study did not include a non-*C. auris* comparison group and did not perform multivariable adjustment, *C. auris* cannot be identified as an independent risk factor for mortality. Previous multicenter and comparative studies have suggested that adverse host factors, ICU stay, central venous catheter use, abdominal surgery, deep-seated candidal complications, inadequate antifungal therapy, mechanical ventilation, diabetes mellitus, chronic kidney disease, malignancy, immunosuppression, and high disease-severity scores may contribute substantially to mortality in candidemia and *C. auris* infection [30,31,32,33]. Our findings are consistent with this interpretation and indicate that mortality should be evaluated in relation to host factors, severity of illness, and treatment timing.

This study has several limitations. First, it was retrospective and single-center, and the sample size was limited, reducing statistical power. Second, molecular typing was not performed; therefore, clade assignment, clonal transmission, outbreak sources, and inter-unit transmission pathways could not be confirmed. Third, environmental sampling and systematic colonization screening were not available. Because standardized prospective criteria were not applied to non-sterile-site specimens, colonization could not be excluded with certainty for some urine or tracheal aspirate isolates, despite clinical management as suspected infection. Fourth, the exact interval from culture positivity to antifungal treatment and the classification of therapy as empirical or targeted were not consistently retrievable; therefore, treatment timeliness and appropriateness could not be assessed. Fifth, AFST was performed using a commercial method, and amphotericin B resistance may have been overestimated. Finally, mortality analyses were exploratory and may have been affected by confounding related to disease severity and underlying comorbidities. Because the cohort included only cases detected through clinically indicated specimens, the study is prone to selection bias and may not reflect the true burden of asymptomatic colonization.

## 4. Materials and Methods

### 4.1. Setting, Study Design, and Patient and Isolate Selection

Our hospital, located in Istanbul, is a tertiary-level training and research hospital affiliated with the Ministry of Health. It has 815 beds across 40 wards, including 166 beds distributed among 10 ICUs. On average, the hospital provides services to 8509 outpatients and 223 inpatients daily. In this institution, the anesthesia intensive care unit (AICU) is an adult intensive care unit administered by the Department of Anesthesia and Resuscitation; IMICU denotes the internal medicine intensive care unit, and PCU denotes the palliative care unit.

This retrospective study was approved by the Clinical Research Ethics Committee of Ümraniye Training and Research Hospital on 25 May 2023 (Decision No. 186). All microbiological specimens with *C. auris* growth detected between January 2021 and December 2022 were initially retrieved from the laboratory information management system (LIMS). Patients younger than 18 years were excluded, leaving 33 adult patients for analysis. The patients were hospitalized in the anesthesia ICU (AICU), internal medicine ICU (IMICU), or palliative care unit (PCU). Clinical data were retrieved retrospectively from the hospital information management system (HIMS), and microbiological data were obtained from the LIMS. Multiple isolates from the same patient were retained when they originated from different specimen types or distinct sampling events; patient-level clinical outcomes were analyzed once per patient.

Routine *C. auris* colonization screening was not performed at our hospital. All specimens included in the study were submitted to the clinical microbiology laboratory because of suspected infection, and the recovered isolates underwent antifungal susceptibility testing. Candidemia was classified when *C. auris* was recovered from at least one blood culture. During retrospective review in collaboration with the treating clinicians, none of the 33 patients was documented as having colonization alone, and all were clinically managed as infected cases. Nevertheless, for isolates from non-sterile sites, particularly urine and tracheal aspirates, colonization could not be excluded with certainty because standardized prospective classification criteria and active screening cultures were not available.

Although this study reports on cases from 2021 to 2022, the retrospective analysis was intentionally designed to specifically isolate and document the initial, early-stage emergence of *C. auris* in our institution, serving as a baseline before the subsequent implementation of updated national infection control guidelines.

### 4.2. Patient and Epidemiological Data

Demographic and clinical characteristics, underlying diseases, risk factors, invasive procedures, ICU exposure, antimicrobial and antifungal treatments, culture sources, and outcomes were retrospectively collected from patient records and HIMS. Candidemia was defined as the isolation of *C. auris* from at least one blood culture obtained from the patient. Antifungal data included the agents administered. However, the distinction between empirical and targeted therapy and the exact interval from culture positivity to treatment initiation could not be reconstructed consistently from the retrospective records and were not included in comparative analyses. Length of hospital stay (LOS) was calculated as the total number of hospitalization days.

### 4.3. Microbiological Data

*C. auris* isolates were identified using matrix-assisted laser desorption ionization–time-of-flight mass spectrometry with VITEK MS v.3.2 (bioMérieux, Marcy-l’Étoile, France). AFST was performed using Sensititre YeastOne YO10 AST Plate kits (Thermo Fisher Scientific, Waltham, MA, USA). AFST results were interpreted according to the CDC interim breakpoints available during the study period [17]. The distribution of isolates by specimen type and hospital ward and the AFST results were evaluated. Molecular clade identification, whole-genome sequencing, or microsatellite typing was not performed; therefore, epidemiological interpretations regarding transmission were made cautiously and were based only on temporal, ward, and culture-source distributions.

### 4.4. Statistical Analysis

Data analysis was performed using Python (v3.10) with the Pandas and SciPy.stats libraries. Continuous variables were presented as mean ± standard deviation (SD) or median (interquartile range), as appropriate. Categorical variables were presented as numbers (n) and percentages (%). The Mann–Whitney U test was used to compare continuous variables between survivors and non-survivors. In the exploratory APACHE II analysis, baseline APACHE II scores and APACHE II-based expected mortality were compared between operative and non-operative patients using two-sided Mann–Whitney U tests; observed mortality by operative status was compared using a two-sided Fisher’s exact test. Age was additionally categorized using a 65-year threshold. Fisher’s exact test was used for other categorical comparisons, and the chi-square goodness-of-fit test was used to evaluate seasonal case distribution. A *p*-value < 0.05 was considered statistically significant. Because of the limited sample size and high mortality rate, mortality-related analyses were considered exploratory and hypothesis-generating rather than confirmatory; multivariable modeling and formal APACHE II calibration analyses were not performed.

## 5. Conclusions

This study describes early institutional *C. auris* cases in a tertiary hospital in Istanbul and shows that cases occurred mainly among elderly, critically ill patients with ICU exposure, invasive devices, prolonged hospitalization, and broad-spectrum antimicrobial use. The findings emphasize the need for rapid laboratory identification, timely initiation of appropriate antifungal treatment, cautious interpretation of commercial AFST results, particularly recognizing the potential for amphotericin B resistance overestimation, strengthened infection control practices, and diligent antifungal stewardship. Future multicenter studies incorporating molecular typing, standardized broth microdilution methods, environmental sampling, and comparison groups are essential to differentiate true clonal transmission from spatiotemporal clustering, and to further elucidate resistance patterns and mortality determinants of *C. auris* infections in Türkiye.

## Figures and Tables

**Table 1:** Demographic and clinical characteristics of the patients (patient-level analysis, n = 33).

Characteristics	n (%) or Mean ± SD
Age (years), Mean ± SD	67.8 ± 18.1
Age ≥ 65 years	20 (60.6%)
Age < 65 years	13 (39.4%)
Gender	
Female	19 (57.6%)
Male	14 (42.4%)
Primary Admission Diagnosis	
Sepsis	13 (39.4%)
Respiratory system diseases	11 (33.3%)
Postoperative complications	7 (21.2%)
Other	2 (6.1%)
Hospital Unit (at the time of first *C. auris* isolation)	
AICU	13 (39.4%)
IMICU	16 (48.5%)
PCU	4 (12.1%)
Comorbidities	
Hypertension (HT)	20 (60.6%)
Diabetes mellitus (DM)	13 (39.4%)
Cerebrovascular event (stroke)	7 (21.2%)
Acute/chronic renal failure	7 (21.2%)
Malignancy	7 (21.2%)
Acute/chronic heart failure	5 (15.2%)
Chronic obstructive pulmonary disease/pneumonia	3 (9.1%)
Coronary artery disease	1 (3.0%)

**Table 2:** Invasive procedures, therapeutic interventions, and other risk factors (patient-level analysis, n = 33).

Invasive Procedures	n (%)
Urinary catheter	32 (97.0%)
Central venous catheter	30 (90.9%)
Mechanical ventilation	27 (81.8%)
Intubation	19 (57.6%)
Tracheostomy	11 (33.3%)
Presence of a drain	7 (21.2%)
External ventricular drainage (EVD)	2 (6.1%)
Renal replacement therapy (RRT)	3 (9.1%)
Length of hospital stay (LOS)	72.73 ± 54.70 days
**Therapeutic risk factors**	
Use of inotropes (at least 1 agent)	24 (72.7%)
Steroid use	9 (27.3%)
Broad-spectrum antibiotic use	33 (100%)
Use of ≥2 antibiotic classes	28 (84.8%)
COVID-19 positivity	1 (3.0%)

**Table 3:** MIC distribution of *C. auris* isolates (isolate-level analysis, n = 62).

Antifungal	MIC Distribution, mg/L (n)	Total Tested (n)	MIC Range (mg/L)
AMB	0.25 (1); 2 (7); 4 (8); 8 (29); ≥16 (17)	62	0.25–≥16
FLC	8 (1); 16 (6); 32 (44); 64 (2); 128 (5); ≥128 (2); 256 (2)	62	8–256 *
MFN	0.06 (14); 0.12 (35); 0.25 (6); 0.5 (1); 1 (1); 8 (4)	61	0.06–8
CAS	0.06 (2); 0.12 (6); 0.25 (49); 1 (1); 4 (1); 8 (3)	62	0.06–8
VOR	0.06 (1); 0.12 (12); 0.25 (26); 0.5 (16); 1 (6); 4 (1)	62	0.06–4

MIC values are reported in mg/L; numbers in parentheses indicate isolate counts. One micafungin result was unavailable. * For fluconazole, two isolates were recorded as ≥128 mg/L and two as 256 mg/L. Resistance was interpreted according to the CDC interim breakpoints available during the study period.

**Table 4:** Descriptive clinical factors associated with mortality (patient-level analysis, n = 33). * *p* < 0.05; † overall Fisher–Freeman–Halton exact test across the three antifungal-therapy categories.

Variables	Survivor n (%)	Deceased n (%)	*p*-Value
**Demographic**			
Age ≥ 65 years, n (%)	4 (44.4%)	16 (66.7%)	0.425
Female sex, n (%)	4 (44.4%)	15 (62.5%)	0.442
**Comorbidity Burden**			
≥3 comorbid diseases, n (%)	3 (33.3%)	12 (50.0%)	0.458
**Invasive Procedures and Interventions**			
Mechanical ventilation (MV), n (%)	5 (55.6%)	22 (91.7%)	**0.034** *
Inotrope requirement, n (%)	4 (44.4%)	20 (83.3%)	0.073
**Microbiological Parameters**			
Positive blood culture (Candidemia), n (%)	7 (77.8%)	18 (75.0%)	1.000
**Antifungal Therapy**			
Echinocandin use, n (%)	8 (88.9%)	13 (54.2%)	0.280 †
FLC and/or AMB use, n (%)	0 (0.0%)	5 (20.8%)	
No antifungal therapy, n (%)	1 (11.1%)	6 (25.0%)	

**Table 5:** Baseline APACHE II scores and expected versus observed mortality by operative status (patient-level exploratory analysis, n = 33). Between-group *p*-values were 0.815 for baseline APACHE II score and 0.300 for APACHE II-based expected mortality (two-sided Mann–Whitney U tests), and 0.309 for observed mortality (two-sided Fisher’s exact test). No formal calibration analysis was performed.

Patient Group	n (%)	Mean Age ± SD	Baseline APACHE II Score, Mean ± SD	APACHE II-Based Expected Mortality, Mean % ± SD	Observed Deaths, n (%)
Operative	6 (18.2%)	72.1 ± 7.3	25.3 ± 5.9	42.2 ± 25.1	3 (50.0%)
Non-operative	27 (81.8%)	66.8 ± 19.7	25.1 ± 4.3	49.3 ± 14.1	21 (77.8%)
Overall	33 (100%)	67.8 ± 18.1	25.2 ± 4.6	48.0 ± 16.4	24 (72.7%)

## Data Availability

De-identified data supporting the reported results are provided in the article and its Supplementary Materials. Additional patient-level clinical records are not publicly available due to privacy and ethical restrictions but may be available from the corresponding author upon reasonable request and subject to institutional approval.

## Supplementary Materials

The following supporting information can be downloaded at https://www.mdpi.com/article/10.3390/antibiotics15090824/s1, Table S1, Master clinical dataset—demographics, comorbidities, interventions, and outcomes (n = 33); Table S2, Antifungal susceptibility profiles and minimum inhibitory concentrations of the 62 *Candidozyma auris* isolates.

## Abbreviations

AFST	Antifungal susceptibility testing
AICU	Anesthesia intensive care unit
AMB	Amphotericin B
CAS	Caspofungin
FLC	Fluconazole
HIMS	Hospital information management system
IMICU	Internal medicine intensive care unit
LIMS	Laboratory information management system
LOS	Length of hospital stay
PCU	Palliative care unit
